# Self make-up: the influence of self-referential processing on attention orienting

**DOI:** 10.1038/srep14169

**Published:** 2015-09-22

**Authors:** Shuo Zhao, Shota Uono, Sayaka Yoshimura, Motomi Toichi

**Affiliations:** 1 Faculty of Human Health Science, Graduate School of Medicine, Kyoto University, Kyoto, Japan; 2 Organization for Promoting Developmental Disorder Research, Kyoto, Japan; 3 Department of Neurodevelopmental Psychiatry, Habilitation and Rehabilitation, Graduate School of Medicine, Kyoto University, Kyoto, Japan

## Abstract

For humans, both eye gaze and arrows serve as powerful signals for orienting attention. Recent studies have shown important differences between gaze and arrows in attention orienting; however, the mechanisms underlying these differences are not known. One such mechanism may be self-referential processing. To investigate this possibility, we trained participants to associate two cues (a red and green arrow in Experiment 1A and two different faces in Experiment 1B) with distinct words (“self” and “other”). Then, we manipulated two types of sound (voice and tone) as targets to investigate whether the cueing effect to self- and other-referential cues differs in a manner similar to that reported for gaze and arrows. We found that self-, but not other-, referential cues induced an enhanced cueing effect to the voice target relative to the tone target regardless of the cue characteristic (i.e., biological or non-biological). Our results suggest that the difference between gaze and arrows in orienting attention can be explained, at least in part, by the self-referentiality of gaze. Furthermore, in Experiment 2, we found a reverse cueing pattern between gaze and arrow cues by manipulating subjects’ experiences, suggesting that differences in the self-referentiality of gaze and arrow cues are not inherent.

Humans are able to process biological signals rapidly. Several lines of evidence suggest that eye gaze is particularly salient and helps us to identify another person’s focus and enables the understanding of other people’s thoughts, beliefs, and desires^1^. Recent studies have shown that infants can orient their attention in the direction of an adult’s gaze and respond appropriately to the cued targets^2^. This finding indicates that the ability to follow another’s gaze, a precursor to theory of mind, has a communicative role before the development of language.

Previous studies have shown that eye gaze reflexively orients the observer’s attention (See^3^ for a review). For example, Friesen and Kingstone (1998)^4^ presented non-predictive gaze cues at the centre of a screen prior to presentation of a peripheral target (right or left). They found that subjects detected the target more quickly when it appeared in the same direction as the cue. Although some studies have found attention orienting differences between gaze and arrow cues^5^,^6^, others have reported similar shifts in attention orienting, regardless of whether the cue was non-biological (arrow) or biological (gaze)^7^,^8^,^9^. Based on these findings, Birmingham and Kingstone (2009)^10^ hypothesised that the apparent difference in attention orienting between gaze and arrow cues could be distinguished only when the cues were embedded in a rich environment. This hypothesis was supported by Zhao *et al.* (2014)^11^ who found that attention orienting by gaze differed from attention orienting by arrows only under a randomised condition, with no difference in responses under a block condition. That is, when these cues were presented randomly, gaze but not arrow cues had a greater cueing effect (i.e., faster responses to a target appearing in a validly cued location than to targets appearing in an invalidly cued location) when the target was a voice versus a tone. In contrast, the enhanced cueing effect of voice was similar when the gaze and arrow cues were presented separately in two blocks. These findings may reflect differences in the relative importance of biological and non-biological stimuli under randomised cue conditions.

However, it remains unclear why gaze and arrows induce different patterns of the cueing effect. The current study aimed to investigate how self-referential processing modulates the cueing effect of gaze and arrows. In contrast to an arrow, another person’s eye gaze is considered a signal of referential evaluation in social interactions, indicating “the degrees to which others regard their relationship with the individual as valuable, important, or close”^12^. For example, when an individual becomes aware of others’ directing their attention at him, he may experience a positive sense of social inclusion, whereas being subjected to averted eye gaze may elicit the impression of a negative relational evaluation, signalling social exclusion. This phenomenon may facilitate rapid detection of a face with direct relative to one with averted gaze^13^,^14^,^15^,^16^, enhancing semantic memory processing^17^. Furthermore, the aversion of eye gaze from an individual is associated with reduced self-esteem, lower feelings of belonging, greater negative mood, and a tendency to infer less positive personality traits about the gaze averter relative to an individual providing direct gaze^18^. Based on these findings, the degree of self-referentiality is one dimension of difference between processing gaze and processing arrows. Additionally, previous studies have demonstrated that self-referential information is processed more effectively than other-referential information is^19^,^20^,^21^ and that gaze direction in self-resembling faces enhances attention orienting compared with faces that do not resemble the self^22^. Thus, we hypothesised that the self-referential nature of eye gaze may explain the difference in the cueing effect between gaze and arrow cues.

In the current study, we first manipulated the self-referentiality of cue stimuli. Sui *et al.* (2009)^23^ developed the following technique to manipulate the self-referentiality of cues: participants were trained to associate a specific arrow shape with themselves, treating it as a self-referential cue, and a different arrow shape with a friend, serving as an other-referential cue. Using a modified version of the method used by Sui *et al.* (2009)^23^, we first trained participants to associate two cues (a red and green arrow in Experiment 1A and two different faces in Experiment 1B) with the words “self” and “other”. We examined whether self- and other-referential cues would show a cueing pattern similar to that reported by Zhao *et al.* (2014)^11^ for gaze and arrow cues. Thus, in Experiment 1A, we predicted that the cueing effect to a voice target would be enhanced by a temporarily established self-referential, but not other-referential, arrow cue. In Experiment 1B, we predicted that the enhanced cueing effect to a voice target would be inhibited by temporarily established other-referential, but not self-referential, gaze cues.

Second, to determine whether the difference in self-referential aspects of eye gaze and arrow cues was experiential or inherent, we directly compared the cueing effect between self-referential arrows and other-referential gaze. In Experiment 2, participants were trained to associate a white arrow with the word “self”, and a face with “other”. We then examined whether the enhanced cueing effect to a voice target would be inhibited by a gaze cue that was temporarily established as other-referential, but not by a self-referential arrow.

## Experiment 1A

### Materials and methods

#### Participants

The research was approved by the local ethics committee of Kyoto University Graduate School and Faculty of Medicine. No foreseeable risk to the participants was present, and personally identifying information was not collected. Participants completed an informed consent form and provided background information. The procedures complied with the ethical standards of the 1964 Declaration of Helsinki regarding the treatment of human participants in research. Twenty naïve students (mean ± SD age, 22.15 ± 3.4 years; 10 males) were recruited for the study and were paid 1,000 yen each. All participants were right-handed as assessed using the Edinburgh Handedness Inventory^24^ and had normal or corrected-to-normal visual and auditory acuity.

#### Stimuli

**Figure 1:**
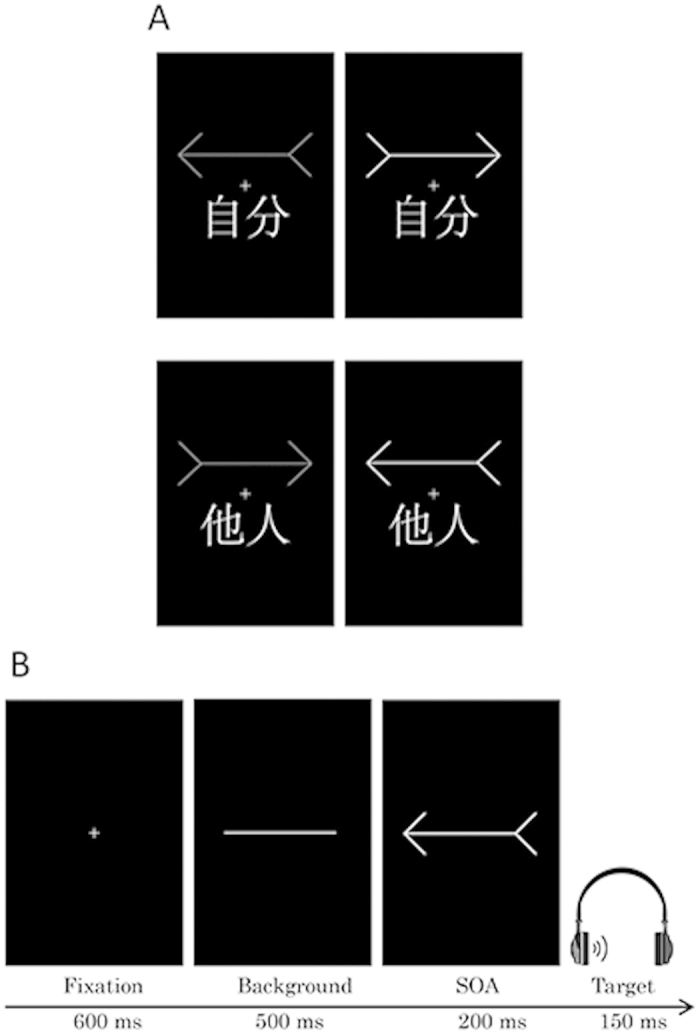
Example stimulus sequence in Experiment 1A. (**A**) Stimuli displayed in the association training session. The actual stimuli were presented in colour (red and green). The white arrow represents the red arrow, and the grey arrow represents the green arrow. (**B**) The stimulus presentation sequence in the arrow-cueing task. SOA, stimulus onset asynchrony.

The stimuli used in the training task are shown in Fig. 1A. A red or green arrow 8.3° wide × 3.0° high was presented above the fixation cross, and the word “self” 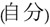 or “other” 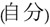, which was 4.3° wide × 3.0° high, was displayed below the fixation cross. The red and green arrows used in the cueing task were the same as those used in the training task (Fig. 1B). As in Zhao *et al.* (2014)^11^, we used two types of auditory stimuli (a voice and a tone) as targets. All stimuli were shown on a black background.

#### Procedure

We conducted two tasks in this experiment. First, we trained participants to associate two arrows (one red and one green) with the words “self” and “other”. Then we used these arrows in the cueing task. After a training block, a cueing block was initiated. All participants performed six blocks consisting of the training and cueing tasks.

#### Training task

Participants were trained to develop an association between self- or other-referential information and the colour of the arrow (Fig. 1A). They were told which colour was associated with “self” and “other”, and assignment of the red or green arrow to the word “self” was counterbalanced across participants. On each trial, a fixation cross was shown at the centre of the screen for 600 ms. Then training stimuli were presented for 100 ms, during which the red or green arrow was presented with the assigned or the unassigned word (“self” or “other”) irrespective of the direction of the arrows. Participants were instructed to respond only when the associated relationship between the arrow and the assigned word was correct by pressing a button as quickly and accurately as possible. Each participant performed six blocks of 64 trials in which all combinations of arrows and words occurred equally in a randomised order.

#### Cueing task

The stimulus presentation sequence is shown in Fig. 1B. For each trial, a fixation cross was presented in the centre of the screen for 600 ms. A neutral stimulus consisting of a transverse white line was then presented for 500 ms followed by a cue stimulus pointing right or left (red or green arrow) in the centre of the screen. The stimulus onset asynchrony (SOA) between the auditory target and the cue was 200 ms. Subsequently, an auditory target stimulus (voice or tone) was presented in the left or right ear for 150 ms through headphones. The participants were instructed to indicate as quickly and accurately as possible whether the target was presented on the left or right side by pressing the corresponding key using their dominant index or middle finger, respectively, on the switch keypad. The temporal resolution of the switch keypad was ∼10 ms. The reaction time (RT) to the target was measured in each trial. The arrow cue remained visible until the response or until 2,150 ms had elapsed. The targets were equally likely to be presented in the same or opposite direction of the cue stimulus. The participants were told that the cues did not predict the target location and were instructed to fixate on the centre of the screen in each trial. The experiment consisted of six blocks of 68 trials including 24 catch trials in which the target did not appear. Forty-eight trials were performed under each condition. Each condition was presented in pseudorandom order. Participants were allowed to rest between blocks. A total of 52 practice trials preceded the experimental trials.

#### Data analysis

For the training task, we measured total error rates (TER), including omission and commission errors, to assess the strength of the association between arrow colour and self- or other-referential words using a cut-off of 10% error in any block. Consistent with a previous study^23^, the participants were required to respond correctly on at least 58 trials in each block. RTs of less than 150 ms or more than 1,000 ms were excluded from the RT analysis (2.14% of the trials). The mean differences in accuracy and RTs between self- and other-referential arrows were calculated for each participant and were analysed using paired *t*-tests.

In the cueing task, incorrect responses (1.4% of the trials) and RTs of less than 150 ms or more than 1000 ms were excluded from the RT analysis (0.48% of the trials). Because the rates of incorrect responses were so low, there was a floor effect for accuracy scores in the experiment. Hence, the error data were not analysed further. The mean differences in RTs between invalid and valid conditions under the cue and target conditions were calculated for each participant. The mean RT differences were analysed using a two-way analysis of variance (ANOVA) with cue (self-referential and other-referential arrows) and target (voice, tone) as the within-participant factors.

### Results and Discussion

#### Training task

The TERs of two participants (one male and one female) were greater than 10% in at least one block and were excluded from the analysis. The results indicated that the association between words (“self” and “other”) and arrow cues was firmly established.

The remaining participants responded significantly more quickly to the arrow associated with “self” than to the arrow associated with “other” (520 ms vs. 535 ms), *t*(17) =3.44, *p* = 0.003, indicating that self-referential information has a higher processing priority than does other-referential information. Accuracy was not significantly different between conditions, *t*(17) =1.53, *p* = 0.145.

#### Cueing task

**Figure 2:**
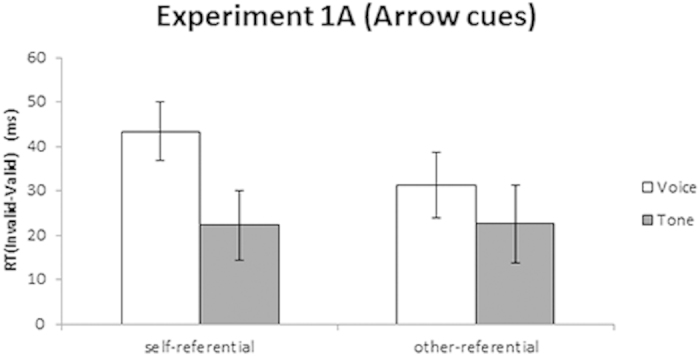
Mean differences in reaction time (RT) between invalid and valid conditions for voice versus tone targets to self- and other-referential arrow cues in Experiment 1A. The error bars represent the standard error (*SE*).

The mean RTs and incorrect responses rates under each condition are shown in Table 1, and the mean differences in RTs between the invalid and the valid conditions for the self- and other-referential cues are shown in Fig. 2. We explored the validity effect under the cue and target conditions using a 2 (Cue: self, other) × 2 (Target: voice, tone) repeated measures ANOVA. The analysis revealed a marginally significant main effect of Target, *F*(1, 17) = 3.66, *p* = 0.07, *η*_*p*_^*2*^ = 0.18; however, we found no significant main effect of Cue, *F*(1, 17) = 0.75, *p*  = 0.399, *η*_*p*_^*2*^ = 0.04. Notably, the Cue × Target interaction was significant, *F*(1, 17) = 4.58, *p*  = 0.047, *η*_*p*_^*2*^ = 0.21. The post hoc test revealed a significantly greater validity effect for voice than for tone under the self-referential (*p*  = 0.023), but not the other-referential, cue condition (*p* = 0.361).

The results of Experiment 1A showed that the enhanced cueing effect to a voice versus a tone target was found only with self-referential cues. This finding suggests that the cueing effect may be mediated by self-referential processing when an association is established between an arrow and self-referential information. In Experiment 1B, we investigated the importance of cue characteristics in self-referential processing by conducting the same task using gaze cues.

## Experiment 1B

### Materials and Methods

#### Participants

A different cohort of 20 naïve subjects (mean age, 21.28 ± 2.42 *SD* years; 11 males) participated in Experiment 1B. All participants provided written informed consent prior to the experiment. A total of 18 participants were right-handed (two were left-handed), as assessed using the Edinburgh Handedness Inventory^24^, and all had normal or corrected-to-normal visual and auditory acuity.

##### Apparatus, design, stimuli, procedure, and analysis

**Figure 3:**
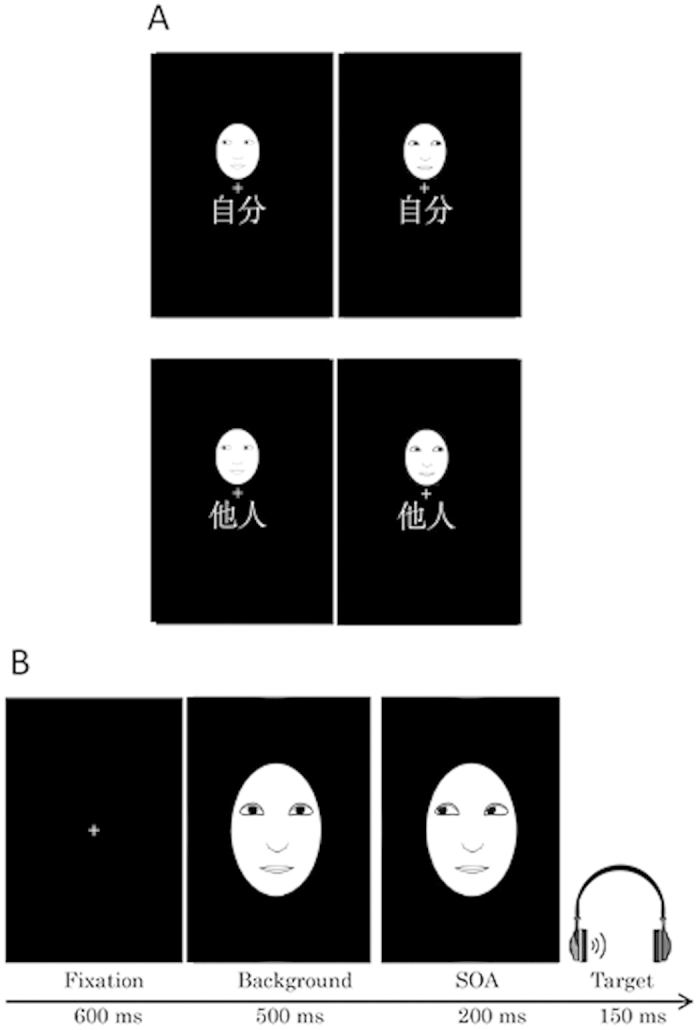
Example stimulus sequence in Experiment 1B. (**A**) Stimuli displayed in the association training session. (**B**) The stimulus presentation sequence in the gaze-cueing task. Actual stimuli were photographs of faces (see Fig. 3 in [25]).

The procedure was the same as that described in Experiment 1A with the exception that two faces, each with an averted gaze (2.8° wide × 4.0° high), were used as the cue stimuli. The face stimuli (JJ and MO) were obtained from Ekman and Friesen (1976)^25^. In the training task (Fig. 3A), RTs of less than 150 ms or more than 1,000 ms were excluded from the analysis (3.65% of the trials). Furthermore, in the cueing task (Fig. 3B), incorrect responses (1.39% of the trials) and RTs of less than 150 ms or more than 1000 ms (0.32% of the trials) were excluded from the analysis. Because the rates of incorrect responses were so low, there was a floor effect for accuracy scores in the experiment. Hence, the error data were not analysed further.

#### Results and Discussion

##### Training task

The TERs of two participants (males) were greater than 10% in at least one block and were excluded from the analysis. Thus, 18 participants were included in the analysis. Participants responded significantly more quickly to a face associated with “self” than to one associated with “other” (571 vs. 605 ms), *t*(17) =5.11, *p* < 0.001, although accuracy did not differ significantly between conditions, *t*(17) =1.41, *p*  = 0.177. These findings were consistent with those in Experiment 1A in that the association between the words (“self”, “other”) and faces was firmly established, and self-referential information showed higher processing priority than did other-referential information.

##### Cueing task

**Figure 4:**
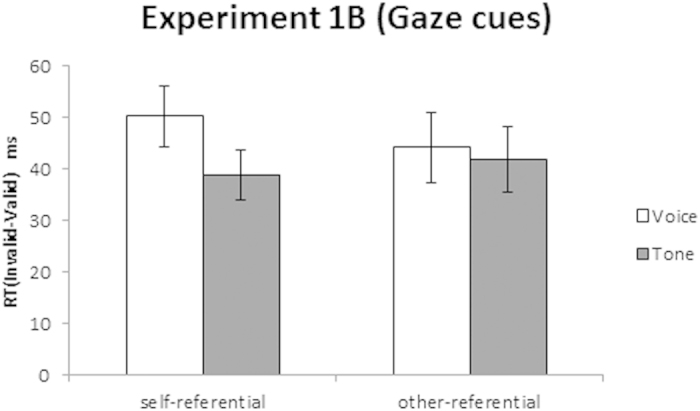
Mean differences in reaction time (RT) between invalid and valid conditions for voice versus tone targets to self- and other-referential gaze cues in Experiment 1B. The error bars represent the standard errors (*SE*).

The mean RTs and incorrect responses rates under both conditions are shown in Table 2, and the mean differences in RT between the invalid and the valid conditions are shown in Fig. 4. We further investigated the effect of validity on the cue and target conditions using a 2 (Cue: self, other) × 2 (Target: voice, tone) repeated-measures ANOVA. The analysis revealed a significant main effect of Target, *F*(1, 17) = 5.56, *p* = 0.031, *η*_*p*_^*2*^ = 0.25; however, the main effect of Cue was not significant, *F*(1, 17) = 0.09, *p* >= 0.767, *η*_*p*_^*2*^ = 0.005. Notably, the Cue × Target interaction was significant, *F*(1, 17) = 5.04, *p* = 0.038, *η*_*p*_^*2*^ = 0.23. The post hoc test revealed that the validity effect was significantly greater for voice than for tone under the self-referential (*p* = 0.001) but not the other-referential gaze cue condition (*p* = 0.575). We found no significant interaction between participant gender and the gender of the face stimuli, which were either female or male (*p* > 0.1).

The results indicate that the cueing effect of a voice versus a tone target was enhanced under the self-referential but not the other-referential gaze cue condition. These findings provide additional evidence suggesting that orienting to a gaze cue is mediated by self-referential processing.

**Combined analysis of Experiments 1A and 1B**. The results of Experiments 1A and 1B were compared directly by a three-way repeated-measures ANOVA on the cueing effect, with Cue (self, other) and Target (voice, tone) as within-participant factors, and Experiment (1A, 1B) as the between-participant factor. We found a significant main effect of Target (*F*(1, 34) = 8.83, *p* = 0.005, *η*_*p*_^*2*^ = 0.21); however, the main effect of Experiment was not significant (*F*(1, 34) = 2.90, *p* = 0.098, *η*_*p*_^*2*^ = 0.08), suggesting that there were no additive effects between self-referential processing and the characteristics of the cue itself. Furthermore, we found no significant interactions between Cue × Experiment, *F*(1, 34) =0.25, *p* = 0.620, *η*_*p*_^*2*^ = 0.01, and Cue × Target × Experiment, *F*(1, 34) = 0.23, *p* = 0.633, *η*_*p*_^*2*^ = 0.01, indicating the absence of interactive effects between self-referential processing and characteristics of the cue itself.

In contrast, the Cue × Target interaction was significant, *F*(1, 34) = 8.64, *p* = 0.006, *η*_*p*_^*2*^ = 0.20. The post hoc tests revealed that the validity effect was greater for voice than for tone targets under the self-referential cue (*p* < 0.001), but not the other-referential cue (*p* = 0.188). These findings indicate that self-referential cues were preferentially associated with a voice target regardless of the cue characteristics (biological or non-biological).

## Experiment 2

Experiment 1 demonstrated that the cueing effect by gaze and arrow cues may be modulated by self-referential processing regardless of the cue characteristics. Zhao *et al.* (2014)^11^ found that gaze, but not arrow, cues had a greater cueing effect when the target was a voice versus a tone. Based on the findings of Experiment 1, we speculated that a reverse pattern of the cueing effect would be found if arrow cues were associated with the word “self” and facial gaze with the word “other”. That is, a greater cueing effect to a voice versus a tone target would be elicited by self-referential arrow cues, but not by other-referential gaze. Given that these two conditions were implemented in different participant groups (Exp 1A and 1B) in Experiment 1, we decided to provide more direct evidence in Experiment 2 that self-referential processing modulates the cueing effect of non-predictive gaze and arrow cues.

### Materials and Methods

#### Participants

In Experiment 2, we tested a different cohort of 35 naïve subjects (mean age, 20.83 ± 1.56 SD years; 18 males) matched with the participants in the combined analysis of Experiment 1. All participants provided written informed consent prior to the experiment. Of the participants, 33 were right-handed (two were left-handed), as assessed using the Edinburgh Handedness Inventory^24^, and all had normal or corrected-to-normal visual and auditory acuity.

#### Apparatus, design, stimuli, procedure, and analysis

The procedure was the same as that described for Experiment 1, with the exception that a white arrow (2.8° wide × 4.0° high) and a female face (MO) with averted eye gaze (2.8° wide × 4.0° high) were used as the cue stimuli. In the training task, we trained participants to associate the white arrow with the word “self”, and the face with the words “other”. RTs of less than 150 ms or more than 1,000 ms were excluded from the analysis (1.93% of the trials). Furthermore, in the cueing task, incorrect responses (1.19% of the trials) and RTs of less than 150 ms or more than 1000 ms (0.33% of the trials) were excluded from the analysis. Because the rates of incorrect responses were so low, there was a floor effect for accuracy scores in the experiment. Hence, the error data were not analysed further.

### Results and Discussion

#### Training task

The TERs of all participants were less than 10% in all blocks, and all the data were therefore included in the following analysis. Participants responded significantly more quickly to the arrow associated with “self” than to the face associated with “other” (511 ms vs. 545 ms), *t*(34) =7.32, *p* < 0.001, although accuracy did not differ significantly between conditions, *t*(34) = 0.96, *p* = 0.346. These findings were consistent with those in Experiment 1, in that the association between words (“self”, “other”) and cues was firmly established, and self-referential had higher processing priority than did other-referential information.

#### Cueing task

**Figure 5:**
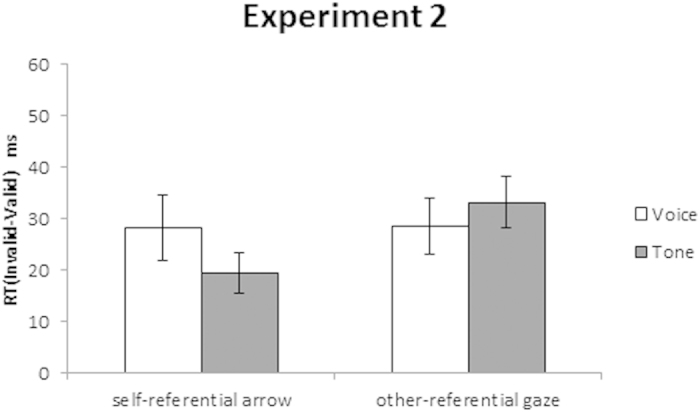
Mean differences in reaction time (RT) between invalid and valid conditions for voice versus tone targets to self- and other-referential gaze cues in Experiment 2. The error bars represent the standard errors (*SE*).

The mean RT and incorrect response rate under each condition are shown in Table 3, and the mean difference in RT between the invalid and the valid conditions is shown in Fig. 5. We further investigated the effect of validity on the cue and target conditions using a 2 (Cue: self-arrow, other-face) × 2 (Target: voice, tone) repeated-measures ANOVA. The analysis revealed that the main effects of Target, *F*(1, 34) = 1.44, *η*_*p*_^*2*^ = 0.41, and Cue were not significant, *F*(1, 34) = 0.35, *η*_*p*_^*2*^ = 0.01; both *p* = 0.239; notably, the Cue × Target interaction was significant, *F*(1, 34) = 11.34, *p* = 0.002, *η*_*p*_^*2*^ = 0.25. The post hoc test revealed a significantly weaker cueing effect for self- than for other-referential cues under the tone (*p* = 0.046) but not the voice target condition (*p* = 0.958). Moreover, the validity effect was significantly greater for voice than for tone under the self-referential arrow cue (*p* = 0.026), but not the other-referential gaze cue condition (*p* = 0.275).

The results indicate that the cueing effect was enhanced for a voice relative to a tone target under the self-referential arrow but not under the other-referential gaze cue condition. These findings suggest that differences in self-referentiality between eye gaze and arrow cues can be determined by experience rather than their being intrinsic to these cue types. As a result of training, effects of orienting by gaze and arrow cues may be mediated by self-referential processing.

## General discussion

The results of the training task showed that participants responded more quickly to the stimuli (arrow or face) associated with “self” than to those associated with “other.” This finding was consistent with previous studies examining the priority of processing of self- versus other-referential information^19^,^20^,^26^. Keyes and Brady (2010)^20^ found that subjects responded more quickly and accurately to their own than to another person’s face. Brédart *et al.* (2006)^27^ showed that self-referential distractors captured attention more readily than other-referential distractors. Our findings indicate that self-referential cues (gaze or arrows) have a higher processing priority than do other-referential cues.

The pattern of the cueing effect in Experiment 1 extends a previous finding that a temporarily established association between self and predictive arrow cues modulates voluntary attention orienting (Sui *et al.*, 2009)^23^. In Experiment 1, after the association between non-predictive cues (gaze/arrow) and words (“self”/“other”) was established, an enhanced cueing effect for a voice target relative to a tone target was clearly observed for self-referential, but not other-referential, gaze and arrow cues. Because participants were not able to predict the location in which a following target would appear, the results indicate that reflexive attention is also modulated by self-referential processing. Moreover, our analysis across Experiments 1A and 1B revealed that cue type (gaze and arrow) did not affect the cueing response. Although previous studies have highlighted the special salience of gaze cues in terms of their biological significance, our findings suggest that self-referential processing also plays an important role in attention in reflexive and voluntary modes.

The present study may also be informative for understanding the differences between eye gaze and arrow cues shown in previous studies; gaze but not arrow cues trigger reflexive attention orienting to counter-predictive cues^5^, induce location-based attention orienting^6^, and show a right-lateralised hemispheric asymmetry for attention orienting^28^. Using the same paradigm as the current study, Zhao *et al.* (2014)^11^ found an enhanced cueing effect for a voice versus tone target under the gaze cue condition, but not under the arrow cue condition. In the present study, self-referential, but not other-referential, gaze and arrow cues triggered an enhanced cueing effect for voice versus tone targets. Combining the results of the two studies, these findings suggest that the difference between gaze and arrows in the cueing effect may be also explained, at least in part, by the self-referential quality of gaze. Furthermore, in Experiment 2, by altering subjects’ experiences (i.e., via a simple training task) to temporarily establish an association between an arrow cue and self-referential information, we were able to elicit the reverse cueing pattern of the one observed by Zhao *et al.* (2014)^11^ for gaze and arrow cues. In Experiment 2, the enhanced cueing effect for a voice versus a tone target was found with the self-referential arrow, but not with the other-referential gaze cue. We suggest that the difference in the self-referentiality of gaze and arrow cues is not inherent and that attention orienting can be moderated by subjects’ experience with different types of cue stimuli.

It is worth addressing the question of why a cueing effect to a voice target is enhanced by a self-referential cue. Previous studies have shown that the cueing effect is enhanced when there is a strong, relevant link between the cue and target, such as when both the cue and the target are biological/social (e.g., gaze cue and voice target)^29^,^11^,^30^,^31^. According to this framework, a gaze cue should induce an enhanced cueing effect to a voice versus a tone regardless of whether self-referential or other-referential words are associated with the gaze cue. However, the current study found that both self-referential arrows and self-referential gaze enhanced cueing to a voice relative to a tone target (Experiment 1). Moreover, we demonstrated that the cueing effect to a voice target was inhibited by other-referential gaze (Experiment 2). To reconcile this contradiction between studies, we speculate that the enhanced cueing effect for a voice target is triggered by the congruence between the cue and the target in terms of self-referentiality. As with a face stimulus, the voice is a special stimulus, commonly called the “auditory face”, and plays a central role in our social cognition^32^. Therefore, a human voice may constitute a target stimulus that is higher in self-referentiality than a pure tone is, as we have argued is the case with facial gaze versus arrow cues. Consistent with this proposition, there is neurobiological evidence^33^,^34^ that voice recognition elicits strong activation in the posterior cingulate gyrus, which is associated with self-referential processing (see^35^ for a meta-analysis). However, further study is needed to determine directly whether congruence of self-referentiality between cues and targets modulates the cueing effect. For example, it would be useful to investigate whether self-referential cues elicit an enhanced cueing effect in response to one’s own name’s sound relative to that in response to another’s name. Additionally, it would be useful to investigate whether arrow cues associated with self-referential information induce the same pattern of cueing effects as gaze cues regardless of the specific situation.

Our findings have implications for understanding impaired social attention in autism spectrum disorder (ASD). Although impairment in attention triggered by social cues (e.g., eye gaze) has been noted in individuals with ASD^36^,^37^,^38^, several experimental studies have found that attention orienting triggered by gaze cues is intact in individuals with ASD^39^,^40^,^41^,^42^,^43^,^44^ for a review, see^45^. Most of these studies investigated attention orienting to gaze and arrow cues separately. However, given the environmental variability inherent in natural settings, the ways in which individuals search for self-referential information may play a critical role in social interactions. Frith and Happé (1999)^46^ argued that the atypical self-awareness characteristic of individuals with ASD reflects a lack of awareness of their own mental state. The finding that self-referential information does not have a high processing priority in individuals with ASD^47^,^48^,^49^,^50^,^51^,^52^,^53^ is consistent with this notion. Investigation of the impact of the self-referentiality of cues on attention orienting in individuals with ASD is a promising area for future research. Use of our paradigm may uncover differences in attention orienting between individuals with and without ASD.

This study has some limitations. In both Experiments 1A and 1B, a difference in RTs between self- and other-referential cues was found when participants responded to a voice as the target under invalid conditions (both, ***p*** < 0.05), suggesting that participants had more difficultly disengaging attention from a voice target with the self- than with an other-referential cue. In contrast, in Experiment 2, a difference in RTs between self- and other-referential cues was found when participants responded to a tone target in valid conditions, suggesting that orienting was delayed for a tone target with the self- compared with the other-referential cue (***p*** = 0.004), whereas they were comparable for the voice target across cues conditions. However, it is unknown whether self-referentiality modulates orienting to or disengagement from a specific target because there is no non-directional condition in the current study. Future research should incorporate a ‘neutral’ baseline, for example, manipulating non-directional cues (e.g., closed eyes and non-directional arrows) rather than contrasting valid trials and invalid trials.

Taken together, Experiments 1A and 1B provide the first evidence for potential mechanisms involved in attention orienting. Our results suggest that the cueing effect may be modulated by self-referential processing. This finding may be invoked to reconsider previously observed differences between biological and non-biological cues in attention orienting in terms of self-referentiality. Furthermore, in Experiment 2, we demonstrated that the cueing effect patterns with non-predictive gaze and arrow cues can be reversed by manipulating subjects’ associations of self-referentiality with these cues, suggesting that differences between gaze and arrow cues with regard to self-referentiality are not inherent.

## Additional Information

**How to cite this article**: Zhao, S. *et al.* Self make-up: the influence of self-referential processing on attention orienting. *Sci. Rep.* **5**, 14169; doi: 10.1038/srep14169 (2015).

## Figures and Tables

**Table 1:** Mean reaction times (ms) in the arrow-cueing task as a function of cue, target, and validity.

Cue and validity		Voice			Tone		
		M	SEM	%E (SD)	M	SEM	%E (SD)
Self-relevant arrow	Valid	368.9	19.4	0.58 (1.6)	381.1	20.5	0.81 (1.5)
	Invalid	412.3	19.3	0.58 (1.6)	403.3	17.6	1.27 (2.0)
Other-relevant arrow	Valid	370.4	17.7	0.93 (2.1)	375.9	19.7	0.93 (1.3)
	Invalid	401.7	17.5	1.27 (2.0)	398.6	17.9	1.39 (1.8)

M, mean; SEM, standard error of the mean; SD, standard deviation; %E, percent error rate.

**Table 2:** Mean reaction times (ms) in the gaze-cueing task as a function of cue, target, and validity.

Cue and validity		Voice			Tone		
		M	SEM	%E (SD)	M	SEM	%E (SD)
Self-relevant gaze	Valid	321.5	10.9	0.58 (2.0)	321.3	9.3	0.93 (2.5)
	Invalid	371.6	12.4	2.43 (4.9)	360.1	11.6	1.62 (3.8)
Other-relevant gaze	Valid	318.5	9.9	0.69 (1.6)	317.7	11.1	0.81 (2.5)
	Invalid	362.5	11.9	1.62 (2.5)	359.4	11.4	2.43 (4.3)

M, mean; SEM, standard error of the mean; SD, standard deviation; %E, percent error rate.

**Table 3:** Mean reaction times (ms) in the gaze-cueing task as a function of cue, target, and validity.

Cue and validity		Voice			Tone		
		M	SEM	%E (SD)	M	SEM	%E (SD)
Self-relevant arrow	Valid	342.7	11.3	1.01 (1.9)	354.8	10.7	0.83 (1.9)
	Invalid	370.9	12.1	0.89 (1.4)	374.2	12.2	1.49 (2.4)
Other-relevant gaze	Valid	344.4	11.9	0.95 (1.6)	342.3	10.8	0.89 (2.0)
	Invalid	372.8	12.0	2.2 (2.6)	375.5	11.8	1.25 (2.4)

M, mean; SEM, standard error of the mean; SD, standard deviation; %E, percent error rate.
